# RCMAT: a regularized covariance matrix approach to testing gene sets

**DOI:** 10.1186/1471-2105-10-300

**Published:** 2009-09-21

**Authors:** Phillip D Yates, Mark A Reimers

**Affiliations:** 1 Department of Biostatistics, Virginia Commonwealth University, Richmond, Virginia 23298, USA

## Abstract

**Background:**

Gene sets are widely used to interpret genome-scale data. Analysis techniques that make better use of the correlation structure of microarray data while addressing practical "n<p" concerns could provide a real increase in power. However correlation structure is hard to estimate with typical genomics sample sizes. In this paper we present an extension of a classical multivariate procedure that confronts this challenge by the use of a regularized covariance matrix.

**Results:**

We evaluated our testing procedure using both simulated data and a widely analyzed diabetes data set. We compared our approach to another popular multivariate test for both sets of data. Our results suggest an increase in power for detecting gene set differences can be obtained using our approach relative to the popular multivariate test with no increase in the false positive rate.

**Conclusion:**

Our regularized covariance matrix multivariate approach to gene set testing showed promise in both real and simulated data comparisons. Our findings are consistent with the recent literature in gene set methodology.

## Background

High-throughput genomic technologies continue to present both rewarding opportunities and novel challenges to biologists and medical researchers. DNA microarray technologies allow researchers to characterize the expression profiles for thousands of genes for samples of interest. However analysis methods for these data are troubled by the "curse of dimensionality" and by small sample sizes due to practical and economic constraints. However because of widespread efforts to arrange genes into meaningful biological subsystems, gene sets, or pathways, have become a widely used unit of analysis. The Kyoto Encyclopedia of Genes and Genomes (KEGG), Gene Ontology (GO), and BioCarta are three widely-used curated resources for warehousing up-to-date gene set information. As researchers move beyond one-dimensional single gene or SNP comparisons researchers will be confronted with a need to compare measures efficiently on functional sets of genes. Subtle differences in expression and co-regulation not detectable with a series of univariate tests may be recovered with a multivariate test. For a complex disease such as cancer this enhancement in statistical power can better equip a researcher to answer the following, "Do the transcription levels for this suspected tumorigenic pathway differ between the normal and cancerous tissue samples?" A more efficient approach to answering this question could lead to improvements in patient treatment regimen or better allow pharmacologists to target their limited resources in the effort to develop novel therapeutic compounds.

Recently a number of tools, either as standalone applications or integrated into a database platform, have appeared to aid in the analysis of gene sets on a genome-wide scale. Tian *et al*. [1] cite the commonplace use of Fisher's exact test (based on the hypergeometric distribution) or its large-sample approximation χ^2 ^form in various software and web tools. PAGE [2], GSEA [3], and GSEA's subsequent refinement [4] are examples of algorithms that combine a set of traditional univariate gene measures into a new composite pathway statistic. One then compares the difference in these composite measures for a family of pathways for the two or more groups under study. Khatri *et al*. [5] and Goeman *et al*. [6] review several issues in the analysis of gene sets. However some difficulties attend approaches based on univariate statistics, such as: if the pathway is formed assuming a set of (at least partially) co-regulated genes then the pathway is intrinsically a multivariate object, the need to apply a multiple comparison correction procedure to the numerous (potentially correlated) single-gene test results to control the overall false alarm rate for the genes within a single pathway comparison, and univariate tests that assume gene independence can lack the power to detect noticeable differences should the genes under comparison be co-regulated. Hotelling's T^2 ^is a classical multivariate test statistic for comparing the difference in multivariate means between two groups which circumvents these difficulties. This test was introduced to the gene-set analysis problem by Kong *et al*. [7]. Song *et al*. [8] compared a similar test to several other popular tests and found that the inclusion of correlation information could improve test quality in their comparison of several gene set analysis methods. Unfortunately the traditional Hotelling's T^2 ^test requires that the number of samples exceeds the number of genes in the set, i.e., n>p, and that a weighted average of the two sample covariance matrices be invertible. In genome-scale microarray studies it commonly occurs that n<p and the combined sample covariance matrix is not invertible and therefore the distribution of the resulting statistic is either unknown or intractable. To circumvent these concerns we have developed a regularized covariance matrix multivariate statistical test (RCMAT) to test for a difference in gene set average transcription levels. In this work we regularize the weighted average of the two sample covariance matrices to guarantee that the resulting matrix is nonsingular. Our measured approach to regularization adapts the familiar Hotelling's T^2 ^test statistic to the ill-posed n<p case.

An early and illustrative application of regularization is ridge regression [9]. In standard linear regression the regression coefficients are estimated using **β**_estimate _= (**X'X**)^-1^**X'Y**. When the **X'X **matrix is ill-conditioned, attributable to high correlations among the regressors and compounded by small sample sizes, the resulting regression coefficient estimates are highly variable. Investigators replace **X'X **by the quantity **X'X **+ k**I **where k is a small constant and **I **is an identity matrix of appropriate dimension. The constant k is generally determined via some heuristic mechanism. In practice in exchange for a small estimator bias a dramatic reduction in estimator variance is often achieved. James-Stein estimators are another well-known class of shrinkage estimators that use a biased estimator to achieve a smaller mean square error for a multidimensional parameter [10]. James-Stein estimators generally assume a (1 - f(**y**))*y form; in general, a simple prescriptive form for shrinkage estimators is not trivial.

Friedman [11] states that regularization techniques are known to be successful in the solution of ill- and poorly-posed inverse problems. Variations of our approach are common in the context of classifying observations via discriminant analysis [11-13] but have not been fully explored for testing the equality of gene sets. A recent application of regularization has also appeared for use in cluster analysis [14], another common microarray analysis tool. Kong *et al*. [7] applied a Hotelling's T^2^-based test; they projected the original data onto a reduced subspace via a singular value decomposition to sidestep the covariance singularity that arises when n<p. Song *et al*. [8] provide a Hotelling's T^2^-based test, PCOT2, which uses a principal coordinate approach to project the original data onto a reduced subspace. In the formulation of our statistic we use permutation testing to determine the significance level of the test statistic. A closed-form solution for the null distribution of our proposed statistic does not exist.

## Methods

### Regularized Covariance Matrix Approach to Testing

The theoretical basis for the RCMAT statistic is the classical Hotelling's T^2^. The standard two-sample form of Hotelling's T^2 ^can be located in [15]. Hotelling's T^2 ^is a scaled distance defined via a positive definite quadratic form, **x'Ax**, for testing H0: **μ1 **= **μ2 **versus H1: **μ1 **≠ **μ2**. For each phenotype group the expression averages and sample covariance matrix of the expression levels within that group is computed for the genes included in the pathway. The two estimated covariance matrices, one for each phenotype group, are combined using the standard pooled covariance estimator, ((n1 - 1) **Σ1 **+ (n2 - 1) **Σ2**)/(n1 + n2 - 2). In the implementation of RCMAT we allow the unpooled covariance estimator to be utilized, if desired. This option can prove useful should the researcher have cause to suspect that differences exist between the two phenotype covariance structures. The inversion of the combined estimate, which is necessary for the test statistic, is ill-posed when n<p. Matrix theory states that the combined estimate must be positive definite and that the eigenvalues of this quantity must be strictly positive [16]. This issue may be addressed by the method of Kong *et al*. [7].

However the estimate of the inverse of the covariance matrix is very unstable when n is only moderately greater than p or large correlations are present in the data. This fact could pose serious problems for the method of Kong *et al*. in many realistic situations. We regularize (shrink) or bias the estimator in the hopes of achieving a more stable estimator, as in ridge regression. The regularized estimate is **Σ**_reg _= α**Σ **+ (1 - α)*I, where **Σ **is the combined covariance estimator obtained with the data and **I **is an identity matrix of the same dimension as **Σ**. Both [10,17] provide an overview of shrinkage estimation, discuss the counterintuitive behaviour of James-Stein estimators, and provide examples in the normal distribution case. **Σ**_reg _could also be viewed as an empirical Bayes estimator; one hopes that through the introduction of bias with the inclusion of **I **a reduction in the total variance of **Σ**_reg _is obtained. Similar to the classification procedure of Tai *et al*. [13] RCMAT employs a heuristic measure to determine the amount of regularization to be applied, comparable to ridge regression. The constant α is incrementally reduced from 1 towards 0, in increments of 0.01, until the smallest positive eigenvalue is greater than one divided by the number of genes in the gene set. Controlling the eigenvalues of **Σ**_reg _insures that the resulting matrix is positive definite. Other approaches to select α are possible. Schäfer *et al*. [18] provide an overview of various shrinkage estimation approaches for use in estimating large-scale covariance matrices in genomics applications. If **Σ **is highly unstable, e.g., the number of genes in the gene set is markedly greater than the sample size or the genes within the gene set are highly co-regulated, then the regularized estimator **Σ**_reg _could be heavily biased towards the identity matrix as α approached 0. That is, the variables are assumed to be nearly independent with unit variance; apart from the biased (co)variance estimates and a reliance on large sample theory this assumption would lead to a test similar to PAGE. Once a suitable α is selected the inverse of the regularized estimate is incorporated into the traditional Hotelling's T^2^.

Despite our efforts to form a stable test statistic with the desired properties we still need the statistic's sampling distribution. A closed form distribution for this test statistic does not exist under the null hypothesis of no average separation between the two phenotypes for the selected gene set. Traditional n>>p large sample asymptotic theory is not applicable due to the customary n<p ill-rank estimate for **Σ**. Therefore, permutation testing [19] is used for assessing significance. 10,000 permutations of the observation phenotype labels were used to determine the significance of the regularized covariance multivariate test. At each permutation step a new α value was selected for the shuffled data. Despite a two-sided hypothesis the use of a quadratic form for our test statistic requires a one-sided rejection region. If one elects to test more than one pathway, i.e., a set of gene sets, then one can apply a multiple comparison procedure to attempt to control for the overall false discovery rate.

### Computer Simulation

A total of 50 conditions were simulated. At each condition 100 data sets were generated. Both the method of Kong *et al*. [7] and the RCMAT algorithm were applied to the simulated data. The expression levels within each phenotype were simulated with a multivariate normal distribution, MVN(**μ**, **Σ**), where **μ **is the vector of gene means and **Σ **is the covariance matrix of the genes within the gene set. With each new data set generation the same random covariance matrix was used for each of the two phenotypes, i.e., phenotype 1 **X **~MVN(**μ1**, **Σ**) and phenotype 2 **Y **~MVN(**μ2**, **Σ**). The phenotype gene set variances were set at one. We did not restrict ourselves to simulating highly structured covariance matrices since we have observed pathways where the pathway members do not exhibit strong pair-wise correlations. Similar observations were noted in Song *et al*. [8] for both diabetes and leukemia data sets. Limited simulation work, not included here, was also performed under the assumption of unequal covariance matrices with similar results. Simulation conditions were intended to reflect typical practice in genomics: 10 or 30 genes were assumed to be in the pathway (p = 10, 30), the within-group phenotype sample size was 10, 20, or 50 (n1 = n2 = 10, 20, or 50), phenotypes were separated on either the major axis of variation (first eigenvector, **e**_1_) or a minor axis of variation (approximately the p/3^rd ^eigenvector, **e**_p/3_), and the amount of separation as a multiple of the axis of variation was either 0.25, 0.5, or 1.0 (c**e**_i _where c = 0.25, 0.5, or 1.0). The aforementioned settings were arranged in a factorial layout. To examine the null distribution of the statistic for possible Type I error concerns we selected 6 conditions (major axis with both 10 and 30 genes at sample sizes of 10, 20, and 50) where no separation existed between the two phenotypes, i.e., **μ1 **= **μ2 **= **0**. Finally, we selected one interesting condition (a pathway comprised of 30 genes where the separation between phenotypes was 0.5 times the major axis) and allowed the within-phenotype sample size to vary at additional increments between 15 and 75.

### GSEA Diabetes Data Sets

We evaluated RCMAT with the human diabetic muscle microarray data found in [3]. Both the transcription data and the gene set data sets from the original GSEA study were obtained from the authors' website (available on-line at http://.www.broad.mit.edu/publications/broad991s). The microarray data included transcriptional profiles obtained from 17 normal and 17 diabetic muscle biopsies. The authors selected 113 of the gene sets for analysis based on their involvement in metabolic pathways with the remainder representing gene clusters based on co-regulated genes from a mouse expression atlas. 22,283 genes were analyzed. Zeros were removed from these data and the logarithm base two transformation was applied to all entries. A median plus/minus three times the median absolute deviation winsorization algorithm was applied to the expression levels of each gene for each phenotype to mitigate the effect of potential outliers. This compares to the "mean plus/minus three standard deviations" recommendation of Draghici [20]. The website contains the 150 gene sets analyzed in the original study. Two gene sets contained a single gene and were removed from the analysis.

### Implementation of RCMAT

RCMAT was written using the freely available language R [21]. R is available for most computing platforms including Windows, MacOS, Linux, and Solaris. The code for RCMAT and generating the simulated data can be found at the author's website (available on-line at http://www.people.vcu.edu/~mreimers/RCMATCode.r).

## Results

### Simulated Data Comparison

**Figure 1:**
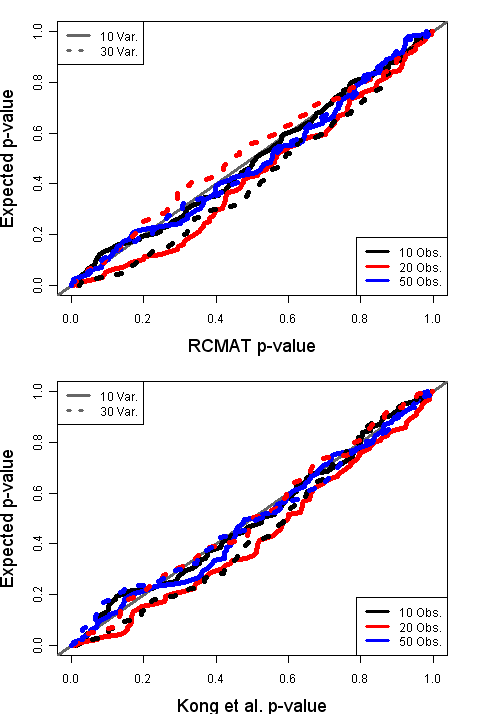
**Null distribution p-values**. Null distribution p-values for both the RCMAT and the method of Kong *et al*. under the null hypothesis (no average difference). P-values for 100 simulated data sets within each of six conditions are given. Along the vertical axis is the expected p-value under the assumption of no difference between the two phenotypes; on the horizontal axis is the corresponding actual p-value obtained from the simulation.

We evaluated the performance of RCMAT with both simulated data and the data from the original GSEA paper [3]. Due to the lack of a benchmark standard for comparing n<p test procedures we chose to compare the performance of our statistic with the approach of Kong *et al*. [7] via simulation. Apart from RCMAT's use of a regularized covariance estimate the method of Kong *et al*. is a close Hotelling's T^2 ^parallel to RCMAT. The Kong *et al*. method was independently presented as PCOT2 in [8]. We first examine the distribution of p-values when no difference exists between the groups in the averages of expression measures of genes in the pathway, i.e., the null hypothesis case. In a two-group null comparison each p-value between 0 and 1 is equally likely. Figure 1 depicts the distribution of p-values for both RCMAT and the procedure of Kong *et al*. when no difference is present between the two groups. Each plot graphs the 100 ranked p-values for each of the six settings in a uniform QQ-plot. The number of genes in a pathway was either 10 or 30 and the within-group sample size was 10, 20, or 50 for both groups. Both methods exhibit a somewhat conservative bias relative to the expected p-value.

**Figure 2:**
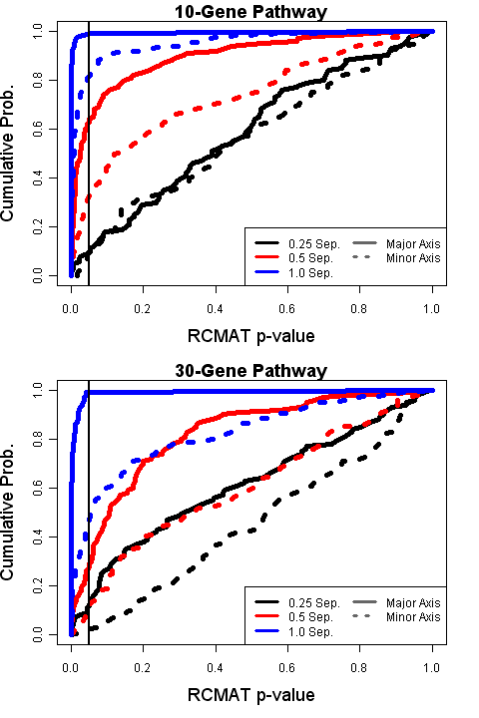
**Cumulative distribution function of RCMAT nominal p-values under several simulated non-null conditions**. Under each of 12 selected conditions 100 simulation experiments were performed and permutation p-values obtained. The vertical axis is the cumulative distribution function, the proportion of values less than the observed p-value, for the 100 simulated data sets within a condition. A vertical line corresponding to a 0.05 nominal p-value is also provided.

**Figure 3:**
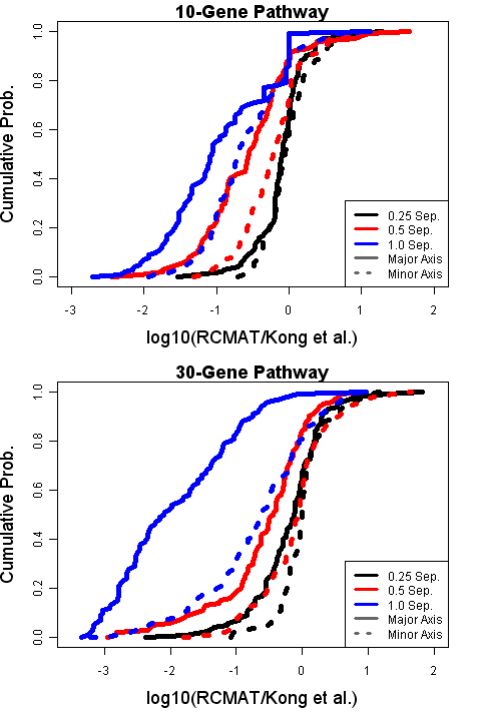
**Cumulative distribution function of a ratio of RCMAT and Kong *et al*. p-values under several simulated non-null conditions**. For 12 non-null conditions 100 simulation experiments were performed and permutation p-values obtained for both the RCMAT and the method of Kong *et al*. The logarithm base ten of the RCMAT p-value/Kong *et al*. p-value ratio is listed on the horizontal axis. The vertical axis is the cumulative distribution function, the proportion of values less than the observed ratio, for the 100 simulated data sets within a condition.

We now turn our attention to the power of the RCMAT approach. Figure 2 illustrates the cumulative distribution function of the RCMAT nominal p-values under select simulation conditions; Figure 3 provides a paired comparison of the RCMAT p-value with the corresponding p-value produced by the method of Kong *et al*. Summary statistics for the 36 non-null simulated conditions can be found in Additional file 1. To facilitate the paired comparison a logarithm base ten ratio (i.e., log_10_(RCMAT p-value/Kong *et al*. p-value) is graphed. Permutation p-values of zero, the only allowable value less than 0.0001, were replaced with 0.00009 in the computation of the ratio.

Figure 2 reveals that our simulation study spans both highly significant to clearly insignificant separations as determined by the median nominal p-value. For presentation we included only those conditions where the within-phenotype sample size was 20. As expected, the real differences between the phenotypes were more easily detected with fewer genes in the gene set (and with larger sample size). See Additional file 1 for complete details. Furthermore the deviations in the minor axis were less easily detected; this may be expected because the regularization modulates the estimate of smaller variances. Figure 2 shows that the median p-value increased from 0.02585 to 0.13425 as the simulation shifted from the major to a minor axis of variation for the p = 10, n1 = n2 = 20, and c= 0.5 condition. The median p-value shifted from 0.13425 to 0.34065 as we increased the number of genes p from 10 to 30 for the same conditions along a minor axis of variation. The first case highlights the difficulty in locating a moderate-sized effect along a non-dominant subspace; the second case highlights the penalty incurred when p is mildly greater than n, p > n.

Figure 3 shows the cumulative distribution functions of the ratio of the RCMAT p-values to the Kong *et al*. p-values for the same data under several different conditions. The 12 non-null conditions included both 10- and 30-gene pathways with 20 observations for each phenotype, the amount of nonzero separation as a multiple of an eigenvector representing the covariance/correlation structure within the gene set was 0.25, 0.5 or 1.0, and the phenotype separation occurs on either the major or a minor axis of variation. The results depend strongly on the number of genes in the gene set; therefore there are separate plots for conditions with 10 and 30 genes in the gene set. For presentation we included only those conditions where the within-phenotype sample size was 20. This illustrates both n>p and n<p cases and comparable results were obtained with other sample sizes. These figures compare the relative power of the two statistics to detect actual separations between two phenotypes without regard to the significance of the p-value/the size of the test. Figure 3 clearly illustrates that the RCMAT p-values can be an order of magnitude or more less than the p-value of Kong *et al*. under several non-null situations. For example, in the 10-gene pathway case approximately one-fifth of the RCMAT p-values were at least one-tenth of the corresponding Kong *et al*. p-value for both a separation of one along a minor axis of variation and a separation of 0.5 along a major axis of variation. Regularization may be necessary in these cases to stabilize the covariance estimator. In the case of 30 genes and a one-unit separation along a major axis, approximately one-half of the RCMAT p-values were at least one hundred times smaller than the corresponding Kong *et al*. p-value. Given the instability of the covariance estimator for the small-sample p ~n ill-rank case the biased estimator was clearly required. The cumulative distribution plots also capture the comparability of the two procedures in some of the less-than-full-rank cases.

RCMAT is a computationally intensive procedure. Computing time is strongly influenced by the number of permutations, the number of genes in the gene set analyzed, the amount of regularization required, and the increment used in selecting the regularization constant α. On a standard single CPU personal computer with a 2.8 GHz microprocessor and 3 GB of computer memory computing times ranged from 2 to 3 hours to process 100 10-gene gene sets using 10,000 permutations and an α increment of 0.01. On the order of 4 to 5 hours of CPU time were necessary to process 100 30-gene gene sets.

### Comparison Using Diabetes Data

Table 1 lists the 50 gene sets (out of 148 tested) with a RCMAT nominal p-value less than 0.05. To facilitate a comparison the p-values determined by the procedure of Kong *et al*. are also listed. Both procedures use permutation testing with 10,000 permutations; hence p-values less than 0.0001 are not attainable. The number of genes in the gene set, which directly affects the quality of the covariance estimator, is also listed. Total computing time for the 148 gene sets was approximately one day. As expected gene sets that contain a large number of genes, roughly one-third of the gene sets analyzed here contained more than 100 genes, took longer to analyze.

**Table 1:** Comparison of RCMAT with the procedure of Kong *et al*.

*Gene Set*	*No. of Genes in Set*	*RCMAT* *p-value*	***Kong et al***. *p-value*
c25_U133_probes	64	0.0003	0.0039
MAP00600_Sphingoglycolipid_metabolism	18	0.0018	0.0036
MAP00300_Lysine_biosynthesis	5	0.002	0.0089
MAP00561_Glycerolipid_metabolism	84	0.0028	0.7803
c29_U133_probes	202	0.0033	0.0672
c33_U133_probes	362	0.0034	0.1201
c23_U133_probes	109	0.0035	0.1581
MAP00360_Phenylalanine_metabolism	23	0.0036	0.0723
MAP00531_Glycosaminoglycan_degradation	18	0.0043	0.0005
MAP00511_N_Glycan_degradation	9	0.0072	0.0066
GLUCO_HG-U133A_probes	46	0.0084	0.4585
GLYCOL_HG-U133A_probes	31	0.0088	0.5699
MAP00910_Nitrogen_metabolism	31	0.0094	0.0385
MAP00430_Taurine_and_hypotaurine_metabolism	12	0.01	0.0888
mitochondr_HG-U133A_probes	615	0.0107	0.05
MAP00650_Butanoate_metabolism	38	0.0109	0.3458
human_mitoDB_6_2002_HG-U133A_probes	594	0.0113	0.0381
c28_U133_probes	288	0.0123	0.1947
MAP00252_Alanine_and_aspartate_metabolism	35	0.0131	0.0472
c20_U133_probes	270	0.0139	0.1125
MAP00190_Oxidative_phosphorylation	75	0.0141	0.2173
c22_U133_probes	194	0.0152	0.016
MAP00710_Carbon_fixation	27	0.0152	0.0297
MAP00340_Histidine_metabolism	32	0.0154	0.2045
MAP00330_Arginine_and_proline_metabolism	63	0.0168	0.0062
c31_U133_probes	346	0.0172	0.3197
MAP00380_Tryptophan_metabolism	88	0.018	0.7238
MAP00380_Tryptophan_metabolism~	88	0.0195	0.7156
c7_U133_probes	349	0.0207	0.1292
c15_U133_probes	264	0.0232	0.4323
MAP00512_O_Glycans_biosynthesis	15	0.0236	0.0322
MAP00970_Aminoacyl_tRNA_biosynthesis	34	0.024	0.1054
c27_U133_probes	266	0.0253	0.1722
MAP00251_Glutamate_metabolism	35	0.0256	0.0259
c12_U133_probes	251	0.0263	0.087
MAP00031_Inositol_metabolism	7	0.0265	0.0677
MAP00410_beta_Alanine_metabolism	27	0.0291	0.5854
c34_U133_probes	452	0.0311	0.2366
c11_U133_probes	192	0.0334	0.4341
c18_U133_probes	248	0.0335	0.0167
MAP00590_Prostaglandin_and_leukotriene_metabolism	34	0.0348	0.1956
c14_U133_probes	302	0.0361	0.1327
c35_U133_probes	470	0.0419	0.2794
OXPHOS_HG-U133A_probes	114	0.0441	0.1705
ROS_HG-U133A_probes	9	0.0446	0.1523
c3_U133_probes	267	0.0455	0.5362
c30_U133_probes	239	0.0462	0.1006
GO_0005739_HG-U133A_probes	227	0.0467	0.3106
MAP00310_Lysine_degradation	35	0.0477	0.4446
FA_HG-U133A_probes	34	0.0485	0.1047

For each of the gene sets from Mootha *et al*. [3] both the RCMAT and the method of Kong *et al*. were applied. Nominal (unadjusted) permutation p-values for each of the two procedures are given. The number of genes in the pathway is also provided.

In the tabled comparison 46 of the 50 ranked RCMAT p-values were less than the p-value produced by the Kong *et al*. method. A direct relationship between RCMAT p-values, obtained through the use of a (potentially strongly) biased covariance estimator, and p-values obtained with a method utilizing subspace projections is complicated by the nature of the test and the features of the data. Across all 148 comparisons the Kong *et al*. algorithm produced 49 p-values less than the corresponding RCMAT p-value. The complete comparison data are listed in Additional file 2. Our approach recovered the oxidative phosphorylation gene set originally found in [3]. Song *et al*. [8] did a detailed comparative analysis of these same data using the sigPathway, GSEA-Category, GSEA-limma, SAFE, GlobalTest, and PCOT2 algorithms. They obtained the largest number of significant gene sets with the GSEA-limma algorithm, using a nominal unadjusted p-value of 0.05. But, the top ranked gene set results varied across the 6 methods tested. Apart from 2 or 3 exceptions, all of the gene sets identified under the various algorithms with a nominal unadjusted p-value of 0.05, including the 10 gene sets determined by GSEA-limma, are a subset of the 50 gene sets located with RCMAT. These results also largely encompass the results obtained with the PAGE [2] approach and the method of [1]. Interestingly enough, applying the Benjamini-Yekutieli false discovery rate procedure with an overall α level of 0.05 indicated that a single pathway, C25_U133_probes, would be declared significant. These results are in broad agreement with the FDR adjusted p-values found in [8] where only one pathway met this cut-off across the 6 gene set analysis algorithms tested.

## Discussion

In traditional n>p two sample multivariate testing problems Hotelling's T^2 ^statistic is commonly used to compare two multivariate means [15]. Two features of this multivariate test are that: 1) the test statistic incorporates the co-regulation or correlation among the features and may provide an improved ability to detect a separation between two multivariate groups that are not distinguishable with any single feature, and 2) all of the mean comparisons are integrated into a single statistical test, which simplifies the problem of multiple comparisons; i.e., there is no need to appeal to a multiple comparison correction procedure within a gene set. Hotelling's T^2 ^has already been used to investigate differentially expressed genes in the two sample case [22]. The multivariable gene set approaches found in [22,23] attempt to resolve the n<p problem through the use of a gene selection procedure. Feature/subset selection approaches often involve intermediate statistical tests and can vary in their use of the researcher's available data. Tomfohr *et al*. [24] use the single largest metagene, obtained with a singular value decomposition of expression values of genes in the group, to compare two phenotype groups. In a related extension Kong *et al*. [7] use a singular value decomposition to locate a reduced gene subspace defined by the eigenvectors whose corresponding eigenvalues exceed a small positive number. However the directions of the subspace corresponding to smaller eigenvalues of **Σ **can be poorly estimated. We conjecture that RCMAT is more powerful relative to the procedure of Kong *et al*. since RCMAT does not restrict the magnitude of the phenotypic transcription differences included and it reduces the noise in the estimate of the covariance matrix, which is inverted. A degree of caution is still advised - highly unstable or p > >n gene set covariance estimators may be heavily biased by RCMAT due to the need for a large amount of regularization.

Both Song *et al*. [8] and Ackermann *et al*. [25] offer a comparison of existing methods for analyzing gene sets via one-dimensional gene enrichment procedures and multivariate comparisons. Song *et al*. [8] found that PCOT2, a Hotelling's T^2^-based test statistic which projects the original data onto a reduced subset via a limited number of principal coordinates, could provide an increased ability to detect altered gene sets through the inclusion of the correlation structure relative to the other gene set methods. They also suggested that including the co-regulation structure can outperform the one-dimensional measures contingent on the features of the data. Large FDR-adjusted p-values were also commonplace in the gene set comparisons. Ackermann *et al*. [25] focused primarily on one-dimensional enrichment procedures although the method of Kong *et al*. was also included in their comparison. In the small sample case they advocated the use of regularization procedures for the tests based on one-dimensional measures. They also noted that the strength of the procedure was impacted by the extent of the correlation structure present in the data. Data comprised of mostly independent features did not appear to benefit from a multivariate procedure relative to the best univariate approaches. Ackermann *et al*. also found that the transformation applied to the one-dimensional measures was critical in the performance of the enrichment procedures. The most accurate transformation tested, a squared transformation, essentially corresponds to a Hotelling's T^2^-test with a diagonal covariance matrix. If co-regulation is a negligible concern in a gene set's definition or is weakly present in experimental data the choice between either a multivariate or an enrichment procedure for testing a gene set's significance is moot.

The regularization methodology employed here is related to the shrinkage methods commonly applied to univariate gene analyses. Shrinkage techniques appear in the Bioconductor package limma [26], SAM [27,28], and its gene set extension SAM-GS [29]. Whereas typical shrinkage methods borrow information from other genes to improve estimates for a single gene our approach shrinks the pooled covariance estimate towards a fixed diagonal covariance matrix. Cui *et al*. [30] and Schäfer *et al*. [18] outline some of the theoretical properties of univariate and multivariate shrinkage estimators, respectively, in the context of microarray studies. Not surprisingly, the use of other forms of regularized covariance estimators is possible. In recent work Tsai *et al*. [31] provide a multivariate analysis of variance test based on Wilks' Λ that makes use of a shrinkage covariance matrix estimator for gene set comparisons. This method applies to the comparison of two or more phenotypes. Regularization towards a diagonal covariance matrix has the benefit of transforming the multivariate distance from the traditional Mahalanobis distance to a Euclidean distance. RCMAT shifts, to the extent necessary, from an explicit and complete use of the co-regulation among the gene set constituents to an independent-genes framework. Should large differences exist between the two phenotype covariance structures the traditional combined estimator can begin to resemble the independent-gene scenario. These points bear mention in addition to the usual comments regarding the numerical stability associated with regularization procedures. Bickel and Levina [32] offer the intriguing finding that a naïve Bayes classifier, which assumes that each pair of genes are conditionally independent, can outperform the classical Fisher's linear discriminant rule in the context of p > n classification problems.

Bickel and Levina [33] provide a related theoretical work outlining the benefits of tapered or banded matrices when the number of variables exceeds the number of observations. Restricting the complexity of the covariance estimator can allow for a more stable estimator. This creates a dilemma for the proposed method between ease-of-use and accuracy for a researcher - restricting the number of unknowns improves accuracy but requires that the gene set structure be precisely defined. Theoretical work on p > n problems, such as [33], often assume that the ratio of the number of variables monitored and the sample size obey a fixed relationship. Definitive guidance for addressing p > >n problems for small-yet-realistic samples, e.g., the GSEA diabetes data presented here investigated gene sets consisting of hundreds of genes with only 34 samples, is still lacking.

## Conclusion

As the era of integrative or systems biology expands researchers' concerns will shift from analyzing single genes to analyzing shifts in coherent sub-systems within the cell. Powerful and well-understood analysis tools will need to be developed to address these challenges. Methods that rely on sophisticated corrections to individual test p-values to screen genes or disregard the correlation structure for a gene set known to consist of co-regulated genes carry risks. In this paper we have outlined a multivariate test statistic that bridges the classical Hotelling's T^2 ^to the current "many genes are measured with a minimal number of samples" environment. Our multivariate statistic parallels the one-dimensional gene shrinkage/enrichment methods currently enjoyed in the microarray analytic lexicon. Despite the simplicity of RCMAT it enjoys favorable limiting traits, resembles a classical "diagonalized" pooled estimator in the event of unequal covariances, and offers greater power relative to a method that reduces the dimensionality of the data using the observed data. The merits of RCMAT have been illustrated via a simulated comparison and verified with the GSEA data. We offer RCMAT as a microarray platform-independent analytical tool for use in the analysis of gene sets.

## Authors' contributions

PDY designed and implemented RCMAT, performed the analyses, and drafted the manuscript. MAR suggested the problem, the approach, supervised the development of RCMAT, and edited the manuscript. All authors read and approved the final manuscript.

## Supplementary Material

Additional file 1 **Summary statistics of the RCMAT nominal p-values under the simulated non-null conditions**. Under each of 36 select conditions (the number of variables/genes defined in the gene set, the sample size of each phenotype, the amount of nonzero separation as a multiple of an eigenvector representing the variance/correlation structure within the gene set, the separation occurs on either the major or a minor axis of variation) 100 simulation experiments were performed and permutation p-values obtained. For each condition various percentiles for the p-values obtained are listed. Click here for file

Additional file 2 **Comparison of RCMAT with the procedure of Kong et al**. For each of the gene sets from Mootha *et al*. [3] both the RCMAT and the method of Kong *et al*. were applied. Nominal (unadjusted) permutation p-values for each of the two procedures are given. The number of genes in the pathway is also provided. Click here for file
