# Supplementary material for: RCMAT: a regularized covariance matrix approach to testing gene sets
Source: BMC Bioinformatics. 2009 Sep 21;10:300. doi: 10.1186/1471-2105-10-300 (PMC3087342; doi:10.1186/1471-2105-10-300)
Supplement: Additional file 1 — Summary statistics of the RCMAT nominal p-values under the simulated non-null conditions. Under each of 36 select conditions (the number of variables/genes defined in the gene set, the sample size of each phenotype, the amount of nonzero separation as a multiple of an eigenvector representing the variance/correlation structure within the gene set, the separation occurs on either the major or a minor axis of variation) 100 simulation experiments were performed and permutation p-values obtained. For each condition various percentiles for the p-values obtained are listed. [file 1471-2105-10-300-S1.DOC]

### Additional file 1.

### File format: DOC (Microsoft Word)

Title: Summary statistics of the RCMAT nominal p-values under the simulated non-null conditions

Description: Under each of 36 select conditions (the number of variables/genes defined in the gene set, the sample size of each phenotype, the amount of nonzero separation as a multiple of an eigenvector representing the variance/correlation structure within the gene set, the separation occurs on either the major or a minor axis of variation) 100 simulation experiments were performed and permutation p-values obtained. For each condition various percentiles for the p-values obtained are listed.

| *Conditions:*  *No. Var., Sample Size, Separation, Axis of Variation* | *Minimum* | *25^th^ Percentile* | *Median* | *75^th^ Percentile* | *Maximum* |
| --- | --- | --- | --- | --- | --- |
| 10, 10, 0.25, Major | 0.0003 | 0.1986 | 0.39605 | 0.653825 | 0.9894 |
| 10, 10, 0.25, Minor | 0.0023 | 0.1206 | 0.48525 | 0.71025 | 0.9867 |
| 10, 10, 0.5, Major | 0.0005 | 0.037275 | 0.15895 | 0.403525 | 0.9982 |
| 10, 10, 0.5, Minor | 0.0011 | 0.09235 | 0.27245 | 0.5116 | 0.9479 |
| 10, 10, 1, Major | 0 | 0.00185 | 0.01095 | 0.07995 | 0.5273 |
| 10, 10, 1, Minor | 0 | 0.0065 | 0.0545 | 0.26365 | 0.9125 |
| 10, 20, 0.25, Major | 0.0001 | 0.176925 | 0.38985 | 0.582775 | 0.9816 |
| 10, 20, 0.25, Minor | 0.016 | 0.13755 | 0.41105 | 0.695575 | 0.9907 |
| 10, 20, 0.5, Major | 0 | 0.0063 | 0.02585 | 0.10385 | 0.9352 |
| 10, 20, 0.5, Minor | 0.0007 | 0.03385 | 0.13425 | 0.5182 | 0.9611 |
| 10, 20, 1, Major | 0 | 0 | 0 | 0.0004 | 0.0588 |
| 10, 20, 1, Minor | 0 | 0.00115 | 0.0076 | 0.0269 | 0.7664 |
| 10, 50, 0.25, Major | 0 | 0.008475 | 0.103 | 0.419125 | 0.9455 |
| 10, 50, 0.25, Minor | 0.0003 | 0.07035 | 0.21795 | 0.464175 | 0.9923 |
| 10, 50, 0.5, Major | 0 | 0 | 0.0001 | 0.0021 | 0.1838 |
| 10, 50, 0.5, Minor | 0 | 0.0076 | 0.02835 | 0.1001 | 0.6884 |
| 10, 50, 1, Major | 0 | 0 | 0 | 0 | 0 |
| 10, 50, 1, Minor | 0 | 0 | 0 | 0 | 0.0058 |
| 30, 10, 0.25, Major | 0.0066 | 0.167475 | 0.41175 | 0.6486 | 0.9953 |
| 30, 10, 0.25, Minor | 0.0023 | 0.2063 | 0.37845 | 0.733425 | 0.998 |
| 30, 10, 0.5, Major | 0.0007 | 0.087875 | 0.24045 | 0.50475 | 0.9918 |
| 30, 10, 0.5, Minor | 0 | 0.156875 | 0.4268 | 0.76055 | 0.9915 |
| 30, 10, 1, Major | 0 | 0.002675 | 0.03175 | 0.097875 | 0.7793 |
| 30, 10, 1, Minor | 0.0007 | 0.066725 | 0.2231 | 0.5667 | 0.9378 |
| 30, 20, 0.25, Major | 0 | 0.0851 | 0.31895 | 0.64625 | 0.9797 |
| 30, 20, 0.25, Minor | 0 | 0.31915 | 0.5442 | 0.836 | 0.9957 |
| 30, 20, 0.5, Major | 0.0001 | 0.040675 | 0.10685 | 0.2591 | 0.9646 |
| 30, 20, 0.5, Minor | 0.0013 | 0.1131 | 0.34065 | 0.6687 | 0.9841 |
| 30, 20, 1, Major | 0 | 0 | 0.0003 | 0.0042 | 0.1479 |
| 30, 20, 1, Minor | 0 | 0.010225 | 0.0567 | 0.255325 | 0.9838 |
| 30, 50, 0.25, Major | 0.001 | 0.0757 | 0.1985 | 0.40815 | 0.9816 |
| 30, 50, 0.25, Minor | 0 | 0.1495 | 0.491 | 0.672625 | 0.9991 |
| 30, 50, 0.5, Major | 0 | 0 | 0.0006 | 0.007 | 0.3489 |
| 30, 50, 0.5, Minor | 0 | 0.05425 | 0.1586 | 0.4016 | 0.9288 |
| 30, 50, 1, Major | 0 | 0 | 0 | 0 | 0 |
| 30, 50, 1, Minor | 0 | 0 | 0.00025 | 0.002175 | 0.2153 |
|  |  |  |  |  |  |
