# Supplementary material for: RCMAT: a regularized covariance matrix approach to testing gene sets
Source: BMC Bioinformatics. 2009 Sep 21;10:300. doi: 10.1186/1471-2105-10-300 (PMC3087342; doi:10.1186/1471-2105-10-300)
Supplement: Additional file 2 — Comparison of RCMAT with the procedure of Kong et al. For each of the gene sets from Mootha et al. [3] both the RCMAT and the method of Kong et al. were applied. Nominal (unadjusted) permutation p-values for each of the two procedures are given. The number of genes in the pathway is also provided. [file 1471-2105-10-300-S2.DOC]

### Additional file 2.

Format: DOC (Microsoft Word)

Title: Comparison of RCMAT with the procedure of Kong et al.

Description: For each of the gene sets from Mootha *et al.* [3] both the RCMAT and the method of Kong *et al.* were applied. Nominal (unadjusted) permutation p-values for each of the two procedures are given. The number of genes in the pathway is also provided.

| *Gene Set* | *No. of Genes in Set* | *RCMAT p-value* | *Kong et al. p-value* |
| --- | --- | --- | --- |
| c25_U133_probes | 64 | 0.0003 | 0.0039 |
| MAP00600_Sphingoglycolipid_metabolism | 18 | 0.0018 | 0.0036 |
| MAP00300_Lysine_biosynthesis | 5 | 0.002 | 0.0089 |
| MAP00561_Glycerolipid_metabolism | 84 | 0.0028 | 0.7803 |
| c29_U133_probes | 202 | 0.0033 | 0.0672 |
| c33_U133_probes | 362 | 0.0034 | 0.1201 |
| c23_U133_probes | 109 | 0.0035 | 0.1581 |
| MAP00360_Phenylalanine_metabolism | 23 | 0.0036 | 0.0723 |
| MAP00531_Glycosaminoglycan_degradation | 18 | 0.0043 | 0.0005 |
| MAP00511_N_Glycan_degradation | 9 | 0.0072 | 0.0066 |
| GLUCO_HG-U133A_probes | 46 | 0.0084 | 0.4585 |
| GLYCOL_HG-U133A_probes | 31 | 0.0088 | 0.5699 |
| MAP00910_Nitrogen_metabolism | 31 | 0.0094 | 0.0385 |
| MAP00430_Taurine_and_hypotaurine_metabolism | 12 | 0.01 | 0.0888 |
| mitochondr_HG-U133A_probes | 615 | 0.0107 | 0.05 |
| MAP00650_Butanoate_metabolism | 38 | 0.0109 | 0.3458 |
| human_mitoDB_6_2002_HG-U133A_probes | 594 | 0.0113 | 0.0381 |
| c28_U133_probes | 288 | 0.0123 | 0.1947 |
| MAP00252_Alanine_and_aspartate_metabolism | 35 | 0.0131 | 0.0472 |
| c20_U133_probes | 270 | 0.0139 | 0.1125 |
| MAP00190_Oxidative_phosphorylation | 75 | 0.0141 | 0.2173 |
| c22_U133_probes | 194 | 0.0152 | 0.016 |
| MAP00710_Carbon_fixation | 27 | 0.0152 | 0.0297 |
| MAP00340_Histidine_metabolism | 32 | 0.0154 | 0.2045 |
| MAP00330_Arginine_and_proline_metabolism | 63 | 0.0168 | 0.0062 |
| c31_U133_probes | 346 | 0.0172 | 0.3197 |
| MAP00380_Tryptophan_metabolism | 88 | 0.018 | 0.7238 |
| MAP00380_Tryptophan_metabolism~ | 88 | 0.0195 | 0.7156 |
| c7_U133_probes | 349 | 0.0207 | 0.1292 |
| c15_U133_probes | 264 | 0.0232 | 0.4323 |
| MAP00512_O_Glycans_biosynthesis | 15 | 0.0236 | 0.0322 |
| MAP00970_Aminoacyl_tRNA_biosynthesis | 34 | 0.024 | 0.1054 |
| c27_U133_probes | 266 | 0.0253 | 0.1722 |
| MAP00251_Glutamate_metabolism | 35 | 0.0256 | 0.0259 |
| c12_U133_probes | 251 | 0.0263 | 0.087 |
| MAP00031_Inositol_metabolism | 7 | 0.0265 | 0.0677 |
| MAP00410_beta_Alanine_metabolism | 27 | 0.0291 | 0.5854 |
| c34_U133_probes | 452 | 0.0311 | 0.2366 |
| c11_U133_probes | 192 | 0.0334 | 0.4341 |
| c18_U133_probes | 248 | 0.0335 | 0.0167 |
| MAP00590_Prostaglandin_and_leukotriene_metabolism | 34 | 0.0348 | 0.1956 |
| c14_U133_probes | 302 | 0.0361 | 0.1327 |
| c35_U133_probes | 470 | 0.0419 | 0.2794 |
| OXPHOS_HG-U133A_probes | 114 | 0.0441 | 0.1705 |
| ROS_HG-U133A_probes | 9 | 0.0446 | 0.1523 |
| c3_U133_probes | 267 | 0.0455 | 0.5362 |
| c30_U133_probes | 239 | 0.0462 | 0.1006 |
| GO_0005739_HG-U133A_probes | 227 | 0.0467 | 0.3106 |
| MAP00310_Lysine_degradation | 35 | 0.0477 | 0.4446 |
| FA_HG-U133A_probes | 34 | 0.0485 | 0.1047 |
| c10_U133_probes | 292 | 0.0535 | 0.1022 |
| MAP00400_Phenylalanine_tyrosine_and_tryptophan_biosynthesis | 12 | 0.0551 | 0.0378 |
| MAP00960_Alkaloid_biosynthesis_II | 4 | 0.0563 | 0.0089 |
| MAP00940_Flavonoids_stilbene_and_lignin_biosynthesis | 4 | 0.0583 | 0.0238 |
| c17_U133_probes | 168 | 0.0611 | 0.0098 |
| c1_U133_probes | 234 | 0.0649 | 0.1044 |
| c32_U133_probes | 295 | 0.0649 | 0.2039 |
| MAP00062_Fatty_acid_biosynthesis_path_2 | 14 | 0.0652 | 0.1106 |
| MAP00350_Tyrosine_metabolism | 47 | 0.0729 | 0.0588 |
| MAP03020_RNA_polymerase | 29 | 0.0748 | 0.3018 |
| MAP00150_Androgen_and_estrogen_metabolism | 28 | 0.0759 | 0.2557 |
| c21_U133_probes | 287 | 0.0784 | 0.1345 |
| MAP00580_Phospholipid_degradation | 10 | 0.0798 | 0.0574 |
| MAP00620_Pyruvate_metabolism | 48 | 0.0864 | 0.414 |
| c26_U133_probes | 326 | 0.0899 | 0.0546 |
| PYR_HG-U133A_probes | 10 | 0.0905 | 0.2631 |
| MAP00471_D_Glutamine_and_D_glutamate_metabolism | 3 | 0.0948 | 0.2048 |
| MAP00950_Alkaloid_biosynthesis_I | 7 | 0.0956 | 0.1278 |
| MAP00361_gamma_Hexachlorocyclohexane_degradation | 55 | 0.0975 | 0.1301 |
| MAP00100_Sterol_biosynthesis | 21 | 0.0986 | 0.5851 |
| c9_U133_probes | 330 | 0.0992 | 0.5634 |
| MAP00602_Blood_group_glycolipid_biosynthesis_neolact_series | 30 | 0.0994 | 0.4115 |
| MAP00230_Purine_metabolism | 159 | 0.1015 | 0.0247 |
| c0_U133_probes | 365 | 0.1093 | 0.3942 |
| MAP00260_Glycine_serine_and_threonine_metabolism | 38 | 0.1095 | 0.2989 |
| MAP00632_Benzoate_degradation | 14 | 0.1185 | 0.1836 |
| MAP00640_Propanoate_metabolism | 31 | 0.124 | 0.0698 |
| c19_U133_probes | 203 | 0.1301 | 0.3119 |
| MAP00533_Keratan_sulfate_biosynthesis | 17 | 0.1404 | 0.2993 |
| MAP00900_Terpenoid_biosynthesis | 11 | 0.1429 | 0.3218 |
| MAP00071_Fatty_acid_metabolism | 102 | 0.1455 | 0.0046 |
| MAP00603_Globoside_metabolism | 17 | 0.151 | 0.1816 |
| MAP00680_Methane_metabolism | 16 | 0.1531 | 0.2257 |
| MAP00272_Cysteine_metabolism | 11 | 0.1534 | 0.0677 |
| MAP00040_Pentose_and_glucuronate_interconversions | 8 | 0.1585 | 0.0817 |
| INS_HG-U133A_probes | 42 | 0.1635 | 0.8081 |
| MAP00500_Starch_and_sucrose_metabolism | 33 | 0.1799 | 0.0776 |
| c2_U133_probes | 288 | 0.1863 | 0.275 |
| MAP00220_Urea_cycle_and_metabolism_of_amino_groups | 29 | 0.1881 | 0.1822 |
| MAP00510_N_Glycans_biosynthesis | 33 | 0.1965 | 0.3312 |
| MAP00860_Porphyrin_and_chlorophyll_metabolism | 35 | 0.1967 | 0.5499 |
| MAP00530_Aminosugars_metabolism | 18 | 0.1994 | 0.5237 |
| MAP00051_Fructose_and_mannose_metabolism | 37 | 0.206 | 0.3794 |
| MAP00010_Glycolysis_Gluconeogenesis | 100 | 0.2146 | 0.2354 |
| MAP00790_Folate_biosynthesis | 20 | 0.2248 | 0.3051 |
| c16_U133_probes | 258 | 0.235 | 0.5414 |
| c5_U133_probes | 245 | 0.2362 | 0.4939 |
| c13_U133_probes | 289 | 0.2471 | 0.1946 |
| c24_U133_probes | 276 | 0.2509 | 0.0197 |
| MAP00450_Selenoamino_acid_metabolism | 12 | 0.257 | 0.403 |
| MAP00271_Methionine_metabolism | 11 | 0.2712 | 0.3141 |
| MAP00532_Chondroitin_Heparan_sulfate_biosynthesis | 12 | 0.2713 | 0.2428 |
| MAP00720_Reductive_carboxylate_cycle_CO2_fixation | 11 | 0.2963 | 0.0384 |
| MAP00240_Pyrimidine_metabolism | 79 | 0.304 | 0.0681 |
| MAP00280_Valine_leucine_and_isoleucine_degradation | 47 | 0.3113 | 0.0699 |
| MAP00030_Pentose_phosphate_pathway | 30 | 0.3145 | 0.4325 |
| MAP00601_Blood_group_glycolipid_biosynthesis_lact_series | 12 | 0.3222 | 0.3947 |
| MAP00020_Citrate_cycle_TCA_cycle | 28 | 0.325 | 0.0633 |
| GLYCOGEN_HG-133A_probes | 27 | 0.3278 | 0.3042 |
| MAP00740_Riboflavin_metabolism | 10 | 0.3379 | 0.1325 |
| c8_U133_probes | 288 | 0.3622 | 0.3425 |
| c6_U133_probes | 272 | 0.3641 | 0.4826 |
| MAP00460_Cyanoamino_acid_metabolism | 14 | 0.3762 | 0.3101 |
| c4_U133_probes | 339 | 0.3881 | 0.2224 |
| MAP00052_Galactose_metabolism | 33 | 0.3952 | 0.0586 |
| MAP00480_Glutathione_metabolism | 34 | 0.4022 | 0.2364 |
| MAP03090_Type_II_secretion_system | 3 | 0.4186 | 0.2969 |
| MAP00472_D_Arginine_and_D_ornithine_metabolism | 9 | 0.4258 | 0.5783 |
| MAP00920_Sulfur_metabolism | 5 | 0.4544 | 0.1158 |
| MAP00550_Peptidoglycan_biosynthesis | 2 | 0.4914 | 0.5978 |
| MAP00061_Fatty_acid_biosynthesis_path_1 | 4 | 0.5091 | 0.2917 |
| MAP00522_Erythromycin_biosynthesis | 5 | 0.5146 | 0.5508 |
| MAP03030_DNA_polymerase | 6 | 0.5161 | 0.347 |
| MAP00253_Tetracycline_biosynthesis | 3 | 0.5189 | 0.2895 |
| MAP00630_Glyoxylate_and_dicarboxylate_metabolism | 14 | 0.5288 | 0.4276 |
| MAP00290_Valine_leucine_and_isoleucine_biosynthesis | 6 | 0.5647 | 0.8803 |
| MAP03070_Type_III_secretion_system | 33 | 0.5758 | 0.6039 |
| MAP00193_ATP_synthesis | 33 | 0.5846 | 0.6031 |
| MAP00195_Photosynthesis | 34 | 0.6174 | 0.0269 |
| MAP00780_Biotin_metabolism | 6 | 0.6778 | 0.4942 |
| MAP00643_Styrene_degradation | 4 | 0.6829 | 0.7469 |
| MAP00625_Tetrachloroethene_degradation | 2 | 0.6859 | 0.6394 |
| MAP00120_Bile_acid_biosynthesis | 35 | 0.693 | 0.0247 |
| MAP00053_Ascorbate_and_aldarate_metabolism | 8 | 0.7022 | 0.7421 |
| MAP00562_Inositol_phosphate_metabolism | 32 | 0.7024 | 0.7072 |
| MAP00631_1_2_Dichloroethane_degradation | 8 | 0.7035 | 0.7435 |
| MAP00140_C21_Steroid_hormone_metabolism | 12 | 0.7185 | 0.6949 |
| MAP00130_Ubiquinone_biosynthesis | 5 | 0.7228 | 0.6338 |
| MAP00440_Aminophosphonate_metabolism | 2 | 0.7234 | 0.3613 |
| MAP00670_One_carbon_pool_by_folate | 31 | 0.7261 | 0.6265 |
| KET_HG-U133A_probes | 8 | 0.7341 | 0.6458 |
| MAP00072_Synthesis_and_degradation_of_ketone_bodies | 7 | 0.7343 | 0.7519 |
| TCA_HG-U133A_probes | 21 | 0.7702 | 0.0191 |
| MAP00520_Nucleotide_sugars_metabolism | 6 | 0.8135 | 0.3718 |
| MAP00770_Pantothenate_and_CoA_biosynthesis | 2 | 0.8215 | 0.8252 |
| MAP00750_Vitamin_B6_metabolism | 3 | 0.8578 | 0.8598 |
| MAP00521_Streptomycin_biosynthesis | 2 | 0.9557 | 0.9762 |
| MAP00760_Nicotinate_and_nicotinamide_metabolism | 10 | 0.9912 | 0.9968 |
|  |  |  |  |
